# Assessment of coronary artery disease wall thickening using phase-sensitive black-blood MRI: initial experience for the evaluation of coronary artery disease

**DOI:** 10.1186/1532-429X-14-S1-P235

**Published:** 2012-02-01

**Authors:** Khaled Abd-Elmoniem, Roderic I Pettigrew, Ahmed Gharib

**Affiliations:** 1 Biomedical and Metabolic Imaging Branch, NIDDK, NIH, Bethesda, MD, USA; 2 NIBIB, NIH, Bethesda, MD, USA

## Background

Coronary arterial wall imaging is a promising non-invasive tool for quantitative assessment of arterial remodeling. Conventional techniques mandate imaging during cardiac rest period as well as nulling the blood signal inside the arterial lumen in order to generate an appropriate lumen-wall contrast. These constraints limited the use of coronary wall imaging. Recently, phase-sensitive dual inversion recovery (PS-DIR) imaging Fetal cardiovascular MRI has been hampered by the lack of a reliable cardiac gating signal. A recently proposed solution to this problem is metric optimized gating (MOG) [1] was proposed to alleviate the burden of nulling the blood signal and permitted more timing flexibility in planning for coronary wall imaging. This work investigates the utilization of PS-DIR in the assessment of coronary artery thickening in patients with coronary artery diseases (CAD).

## Methods

A total of 45 subjects were recruited for this study. These included 23 subjects with calcified and non-calcified plaque as identified on Multi-detector CT angiography (MDCTA) . For controls; 22 subjects without known CAD (confirmed on MDCT) or CAD risk factors (table 1a) were included. A detailed assessment of CAD risk was performed and Framingham Risk Score (FRS) was calculated. All subjects were scanned using PS-DIR for the assessment of coronary wall thickness.

Anatomical slices perpendicular to the proximal part of the right coronary artery (RCA) at end-systole were planned similar to a previously published methodology. The location of imaging was chosen such that there was no visible plaque detected at the CTA. PS-DIR images were acquired with a fixed imaging time (TI) = 200ms after the DIR pulse (slice thickness=8mm, FOV=190x190mm, matrix=320x320, interleaves=20, acq.window=18ms/interleaf). Data were acquired during free breathing using 3mm navigator gating and correction. Images with good and excellent quality, as identified by anonymzed reading, were used for analysis. Vessel SNR, CNR, and sharpness were measured on the signed-magnitude PS-DIR images. Mean vessel wall thickness was measured automatically on the images. Unpaired t-test was utilized to compare between control and patient measurements.

## Results

**Figure 1:**
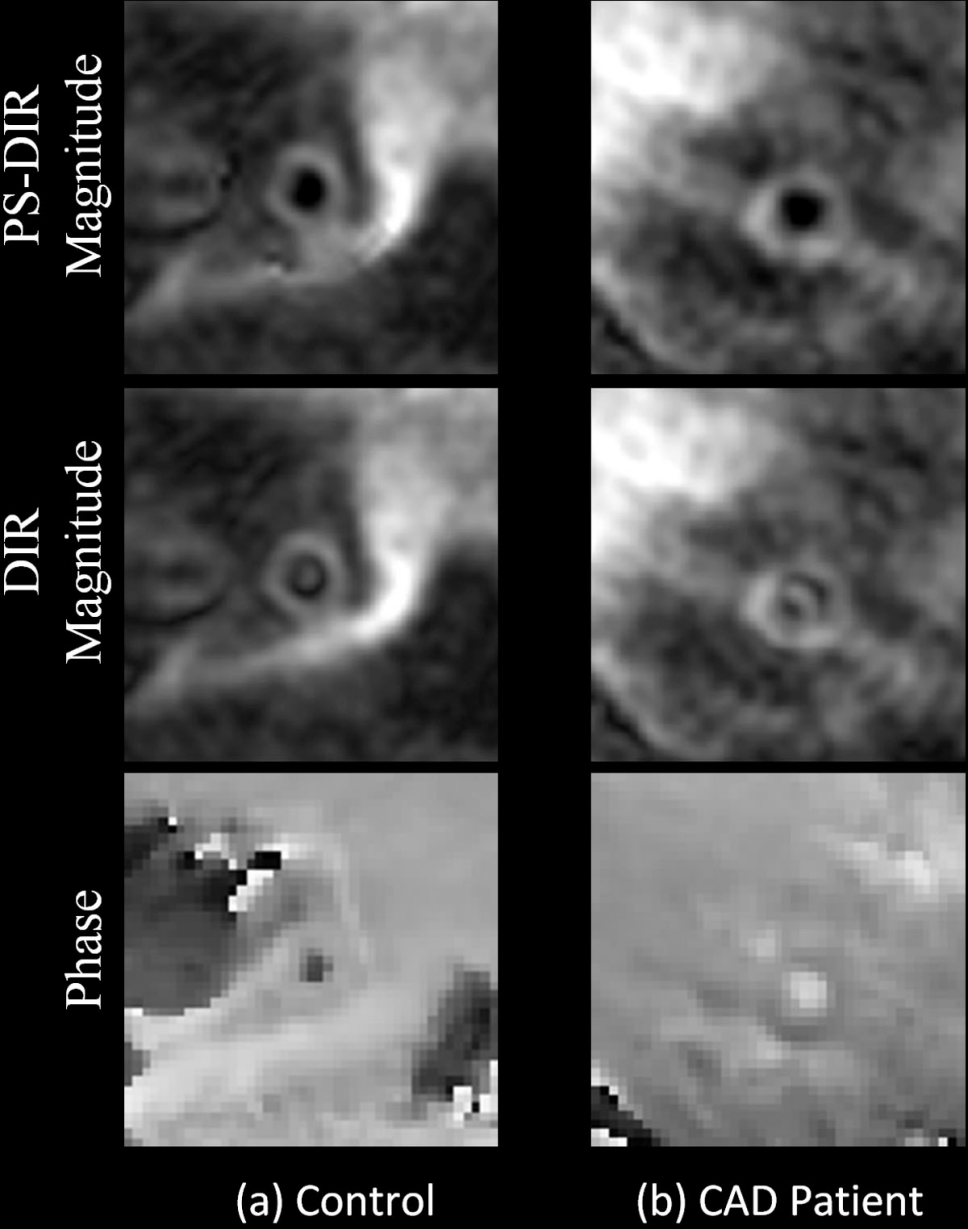
Coronary arterial wall PS-DIR images of normal and patient cases. Signed-magnitude PS-DIR reconstruction successfully restores lumen-wall contrast.

PS-DIR was successfully completed in 76% of the subjects. Failure was due to absence of rest period, bad ECG triggering, or motion artifacts. As shown in table 1b, there was no difference in image CNR and CNR between groups. At the imaged RCA locations without noticeable plaques (Fig.1), coronary wall thickness was thicker in patients in comparison to control subjects (1.67mm vs. 1.24mm, p<0.001).

**Table 1:** 

Study population characteristics. *difference is statistically significant			
	**Patients**	**Normal**	**p-value**

Number (total, males)	23.9	22.8	-
Age (mean±SD) [years]	58±11*	29±6*	<0.001
BMC (mean±SD)	28±4	26±5	0.08
Hypertension (n)	16	0	-
Smoking (n)	4	0	-
HDL (n)	6	0	-
Diabetes (n)	1	0	-
AIC (n)	2	0	-

(b) PS-DIR coronary arterial wall thickness and image quality indexes in normals and patients. *difference is statistically significant			

	**Patients**	**Normal**	**p-value**

Wall thickness (mean±SD) [mm]	1.67±0.23*	1.24±0.15*	<0.001
SNR (mean±SD)	20.1±12.2	21.3±10.6	0.72
CNR (mean±SD)	8.9±5.3	7.2±3.9	0.20
Vessel Sharpness (mean±SD)	31.6±13.3	34.7±16.0	0.46

## Conclusions

This study demonstrates that CAD patients with confirmed plaques have thicker coronary walls at sites without noticeable plaques as confirmed by MDCT. This thickening is successfully measured using PS-DIR with a fixed imaging time TI without the need to adjust imaging parameters or image at the time of blood-signal nulling. PS-DIR is capable of differentiating between subjects with and without known CAD.

## Funding

NIH.
